# Modified Gegen Qinlian Decoction modulated the gut microbiome and bile acid metabolism and restored the function of goblet cells in a mouse model of ulcerative colitis

**DOI:** 10.3389/fimmu.2024.1445838

**Published:** 2024-08-06

**Authors:** Jinke Huang, Jiaqi Zhang, Fengyun Wang, Xudong Tang

**Affiliations:** ^1^ Department of Gastroenterology, Guangdong Provincial Hospital of Chinese Medicine, Guangzhou, China; ^2^ Institute of Digestive Diseases, Xiyuan Hospital of China Academy of Chinese Medical Sciences, Beijing, China

**Keywords:** ulcerative colitis, modified Gegen Qinlian decoction, gut microbiota, bile acids, goblet cells

## Abstract

**Objective:**

Modified Gegen Qinlian Decoction (MGQD) has been shown to effectively relieve ulcerative colitis (UC) without a known pharmacological mechanism. In this study, the anti-colitis efficaciousness of MGQD and its underlying mechanisms in UC were evaluated.

**Methods:**

Mice with colitis were administered MGQD for 7 days. Following the evaluation of clinical symptoms, gut microbiota in the feces of UC mice was examined using 16S rRNA sequencing and bile acids (BAs) were examined using LC/MS. Gut microbiota consumption and fecal microbiota transplantation (FMT) were used to explore the involvement of gut microbiota in the anti-UC action of MGQD.

**Results:**

MGQD relieved colitis as shown by weight loss protection, a lower disease activity index (DAI), restoration of intestinal length reduction, and lower histopathologic scores. MGQD also restored crypt stem cell proliferation and function of colonic goblet cells, and promoted MUC2 protein secretion. Interestingly, investigations using gut bacterial depletion and FMT showed that MGQD attenuated colonic damage in a gut-dependent way. The modulation of the gut microbiota by MGQD might be attributed to a decrease in *Odoribacter* and an increase in n*orank_f_Muribaculaceae*. In addition, MGQD modulated the metabolism of BAs while restoring the structure of the gut microbiota.

**Conclusion:**

MGQD significantly alleviated colitis in mice, which may be associated with the modulation of gut microbiota and BA metabolism and restoration of function of goblet cells. However, factors other than the gut microbiota may also be involved in the amelioration of UC by MGQD.

## Introduction

Ulcerative colitis (UC) is recurring and remitting inflammatory bowel disease (1). The incidence of UC has been reported to increase year by year worldwide (2). Importantly, North America (Canada = 23.1 per 100,000), Australia (17.4 per 100,000), and Northern Europe (Faroe Islands = 57.9 per 100,000) have the greatest incidences of UC, with industrialized countries and areas having greater incidences than developing ones (3). Currently, inducing both clinical and endoscopic remission with long-term maintenance is the aim of UC treatment (4). Medications that contain 5-aminosalicylic acid (5-ASA), corticosteroids and biologics are frequently utilized therapeutic agents (4). Yet, serious side effects and medication resistance are challenges associated with long-term usage of these medications (5, 6). As a result, there is a growing interest among researchers in complementary and adjunctive therapies for UC (7, 8).

The pathological process of UC is intimately linked to the gut microbiota (9, 10). A variety of metabolites, including bile acids (BAs), are regulated by the gut microbiota and act as important signaling factors in the development of UC (9, 10). Individuals with UC exhibit dysbiosis of the gut microbiota, which increases the permeability of the intestinal epithelial cell barrier as well as subsequently causes intestinal inflammation (11). BAs are receiving greater attention than other types of metabolites isolated from the host-gut microbiota due to their physiological importance (12). Intestinal barrier function and enterohepatic detoxification condition of inflammatory bowel disease are reflected in serum BA profile, which highlights the important role that BAs play in the UC process (13). Thus, modifying gut microbiota and BAs to support intestinal mucosal healing and reduce mucosal inflammation has significant therapeutic implications in UC research (14, 15).

Gegen Qinlian decoction is a renowned classical Chinese herbal decoction comprising *Coptis chinensis* French*, Pueraria lobata* (Willd.) Ohwi, *Glycyrrhiza uralensis* Fisch and *Scutellaria baicalensis* Georgi (16). Traditionally, Gegen Qinlian decoction has been used for the treatment of diarrhea with damp-heat syndrome (16, 17). On the basis of traditional application background and theory of damp-heat syndrome, Prof. Xudong Tang added *Zingiber officinale* Roscoe and *Talcum* to Gegen Qinlian Decoction in combination with his decades of clinical practice experience to form MGQD with the aim of improving its effect in the treatment of UC (18, 19). Our previous studies have demonstrated the effectiveness of MGQD in alleviating colitis symptoms and mitigating colonic injury in mice with UC (18, 19). Despite the positive pharmacological benefits of MGQD on UC, the underlying mechanisms have not been elucidated. Therefore, this study sought to clarify potential MGQD therapeutic pathways for UC based on the relationship between gut microbiota and BAs.

## Materials and methods

### Reagents

Proteintech provided the anti-MUC2 (27675-1-AP), anti-SRC (11097-1-AP), and anti-YAP (81090-1-RR) antibodies. Anti-Ki67 (GB111499) was purchased from Servicebio, and anti-Lgr5 (bs-20746R) was purchased from CapitalBio. MP Biomedicals provided the dextran sulfate sodium (DSS). Quanzhou Ruixin Biotech provided mice ELISA kits for TNF-α (RX202412M), IL-6 (RX203049M), and IL-1β (RX203063M).

### Animals

SPF Biotech (Beijing, China) provided C57BL/6 male mice, 4-6 weeks of age and 18–22 g in weight. The mice were kept in rooms with a 12-hour light/dark cycle, a relative humidity range of 40–60% and a temperature maintained between 20 and 24°C. Throughout the acclimatization period, all mice were provided unrestricted access to standard rodent feed and water for one week. The Regulation on the Administration of Laboratory Animals was followed for conducting animal experimental protocols, with approval from the Xi Yuan Hospital’s Animal Ethics Committee.

### Decoction preparation

The MGQD (consisting of *Glycyrrhiza uralensis* Fisch (6 g), *Coptis chinensis* French (9 g), *Scutellaria baicalensis Georgi* (9 g), *Pueraria lobata* (Willd.) Ohwi (24 g), *Zingiber officinale* Roscoe (9* g*) and *Talcum* (9* g*) at a ratio of 2:3:3:3:8:3) was supplied by the pharmacy of Xiyuan Hospital. A voucher specimen (samples NO. 198410132) of MGQD was deposited at the pharmacy of Xi Yuan Hospital. The production process of MGQD extract was as follows: (a) Talcum was cooked in water for 30 minutes, followed by the addition of the remaining herbs and boiling for 1.5 hours before filtration; (b) the filtrate was then mixed with six times distilled water and boiled for 1 hour before filtering again; (c) the resulting filtrate was combined to produce crude drug at a concentration of 2 g/mL.

### Animal experimental protocol

**Figure 1:**
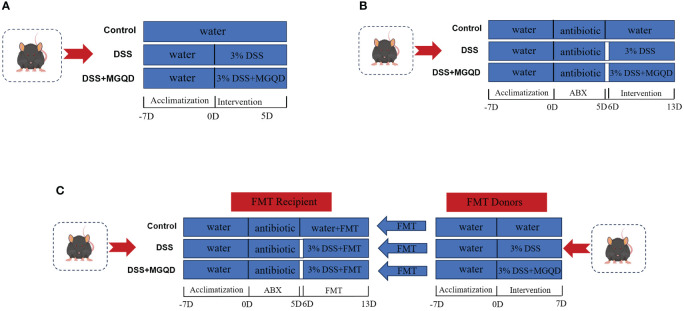
Animal experimental protocols. **(A)** The experimental protocol I, **(B)** the experimental protocol II, **(C)** the experimental protocol III.

The following were the experimental protocols:

- To evaluate the influence of MGQD on mice with UC, 30 mice were separated into three groups: control, DSS, and MGQD-treated UC. For seven days, a 3% DSS solution was given to each of the other groups, excluding the control group. Having compared low, medium, and high doses of MGQD, our previously published studies (18, 19) determined that the high-dose mode of MGQD administration resulted in the most satisfactory improvement in mice with UC, so this current study used high-dose MGQD intervention in mice with UC. Therefore, mice in MGQD group were given MGQD (20 mg/kg) via gavage during the experimental period, while the remaining mice received an equivalent volume of saline. Fresh fecal samples were collected daily from mice in each group under sterile conditions, placed in sterile containers, and stored in a -80°C freezer. The detailed experimental process is depicted in (**Figure 1A**).
- To ascertain the involvement of gut microbiota in the effects of MGQD on mice with UC. Thirty mice were separated into three groups: antibiotic (ABX) control (ABX-control), ABX-disposed DSS (ABX-DSS), ABX disposed MGQD-treated UC (ABX-MGQD). Following our previously established protocol (20), all mice underwent gut microbiota depletion by receiving an ABX cocktail (0.5 g/L neomycin, 0.5 g/L vancomycin, 1 g/L metronidazole, and 1 g/L ampicillin) for 5 days. Our previous study found that a substantial reduction in total DNA levels of intestinal microbiota was observed in the feces of antibiotic-exposed mice compared to control mice, implying that ABX cocktail is effective in eliminating intestinal microbiota in mice (20). Subsequently, the UC mouse model was induced and subjected to MGQD treatment according to the experimental procedures outlined in part I. The detailed experimental process is depicted in (**Figure 1B**).
- To validate the involvement of gut microbiota in the amelioration of UC by MGQD, fecal microbiota transplantation (FMT) experiments were conducted. Thirty mice were separated into three groups: FMT-control, FMT-DSS, and FMT-MGQD. The preparation for gut microbiota depletion and UC induction remained consistent with those outlined in part II. Mice from part I served as donors. For FMT experiments, 1 ml of sterile PBS was added to every 100 mg of collected feces. The mixture was centrifuged for three minutes at 800 rpm after being vortexed and aggressively stirred for ten seconds. Within ten minutes, the supernatant was gathered and given to the recipient mice via gavage, at a rate of 0.1 mL per 10 g of body weight, for seven days in a row. The detailed experimental process is depicted in (**Figure 1C**).

### Histopathological analysis and mucus staining of colon

Paraffin was used to embed colon samples, which were then cut into 5-um-thick slices and stained with periodic acid-Schiff (PAS), alcian blue (AB) and hematoxylin hematoxylin-eosin (HE). Based on previously defined standards, pathologic scoring was carried out (21).

### Measurement of the levels of proinflammatory cytokines

Mice with UC had their colonic tissues’ levels of IL-1β, IL-6 and TNF-α tested using the proper ELISA kits and according to the manufacturer’s recommendations.

### Immunohistochemistry

Ki67-targeting antibodies was used in immunohistochemistry, following the normal methodology. Prior to incubating with the secondary antibody, the samples were first incubated with the primary antibodies for an entire night at 4°C. The stained sample images were taken with a light microscope. Staining intensity was analyzed and represented using the mean optical density value.

### Immunofluorescence staining

Using antibodies that target Lgr5 and MUC2, the conventional approach was followed to perform immunostaining. After exposing the samples to the primary antibodies for an entire night at 4°C, they were rinsed with PBS and then incubated for one hour at 37°C in the absence of light using the corresponding fluorescent secondary antibody. The samples were examined under a fluorescence microscope following DAPI counterstaining.

### Analysis of gut microbiota

Fecal genomic DNA was extracted by E.Z.N.A.^®^ Soil DNA Kit in accordance with standard procedure, and then electrophoresis on 1% agarose gel was used to identify the genomic DNA. The V3–V4 region of the bacterial 16S rRNA genes was amplified by PCR. Following PCR product purification, Quantus™ Fluorometer was used to identify and measure the results. The Illumina NovaSeq PE250 platform was utilized for the sequencing process. To analyze the microbial community structure and taxonomic diversity, the obtained raw reads were processed using Quantitative Insights into Microbial Ecology Version 2 (QIIME2, v2020.2) (22).

### Targeted BAs quantitative analysis

Mix 10 mg of colon contents with 400 μL of methanol and centrifuge at 12000 rpm for 10 minutes. Re-centrifuge after adding 600 μL of methanol to 300 μL of the supernatant. Gather the filtrate for LC-MS analysis after filtering the supernatant using a 0.22 μm membrane. AB SCIEX API 4000 mass spectrometer was used to evaluate the metabolite profiles. The retention period of the target compound on the analytical column was the primary basis for chromatographic characterization. The primary method used in mass spectrometry characterization was high-resolution mass spectrometry, which provided the precise molecular mass of the chemical. Quantification was mainly based on the amount of the standard as the horizontal coordinate, the peak domain of the standard as the vertical coordinate graph to obtain the mathematical relationship between the target compound and its peak area, and then calculate the concentration of BAs in the actual sample based on the peak domain of the corresponding compounds in the unknown sample.

### Statistical analysis

The mean ± standard deviation was used to express all continuous data. Following the completion of Brown-Forsythe test and Shapiro-Wilk normality test, one-way ANOVA analysis was performed if the data met both variance chi-square and normal distribution; in cases where the data were not normally distributed, the Kruskal-Wallis test was utilized; in case the data fit the distribution but not the variance chi-square, Welch one-way ANOVA analysis was utilized; in cases where the data did not match the distribution criteria, the Kruskal-Wallis test was utilized. *P* < 0.05 was used to determine statistical significance for differences. Graph-Pad Prism version 9 and SPSS 26.0 were used for statistical analysis.

## Results

### MGQD attenuated colitis in mice

**Figure 2:**
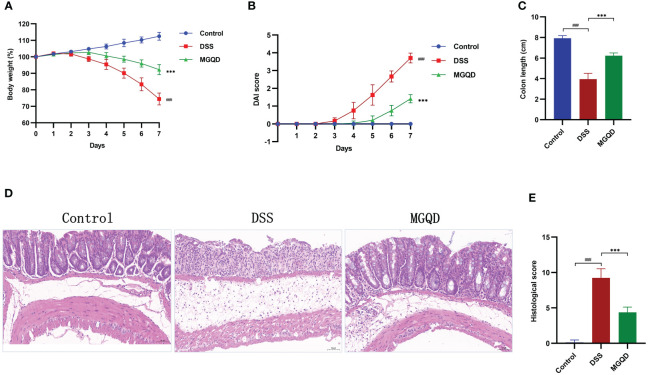
MGQD ameliorated colitis induced by DSS in mice. **(A)** Daily changes in body weight in different groups, **(B)** daily changes in DAI score in different groups, **(C)** comparison of colon length, **(D, E)** representative H&E staining images of colon sections and the comparison of histopathological scores (200× magnification). ###*P* < 0.001 vs. control, ***P < 0.001 vs. DSS.

The DSS group of mice showed reduced body weight, increased disease activity index (DAI), and shorter colon length than the control group, which are typical indicators of colitis (**Figures 2A–C**). Interestingly, following administration of MGQD, colitis symptoms in mice were markedly ameliorated (**Figures 2A–C**).

Pathological results (**Figures 2D, E**) revealed that mice in DSS group exhibited significant colonic tissue damage, characterized by disruption of crypt structure, disorganization of mucosal structure and inflammatory cell infiltration. Surprisingly, MGQD treatment reversed the above pathologic changes and reduced histopathologic scores in mice with UC (**Figures 2D, E**).

### MGQD inhibited proinflammation in mice with colitis

**Figure 3:**
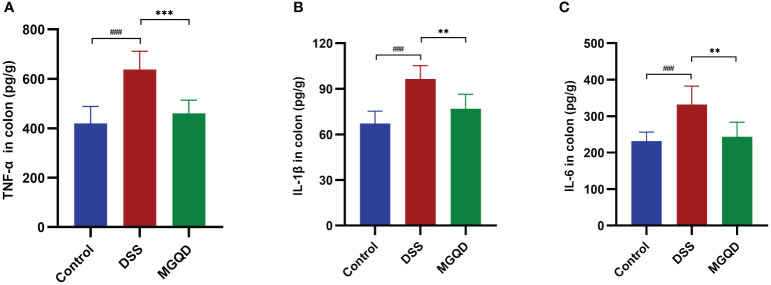
MGQD inhibited proinflammation in mice with colitis. Levels of **(A)** TNF-α, **(B)** IL-1β, **(C)** IL-6. ###*P* < 0.005 vs. control, **P < 0.01 vs. DSS, ***P < 0.001 vs. DSS.

Significantly higher levels of IL-6, IL-1β and TNF-α were detected in mice with colitis compared to control mice (**Figure 3**). After receiving MGQD therapy, these pro-inflammatory cytokines were significantly decreased in mice with UC (**Figure 3**).

### MGQD restored the intestinal mucus barrier by maintaining goblet cell function

**Figure 4:**
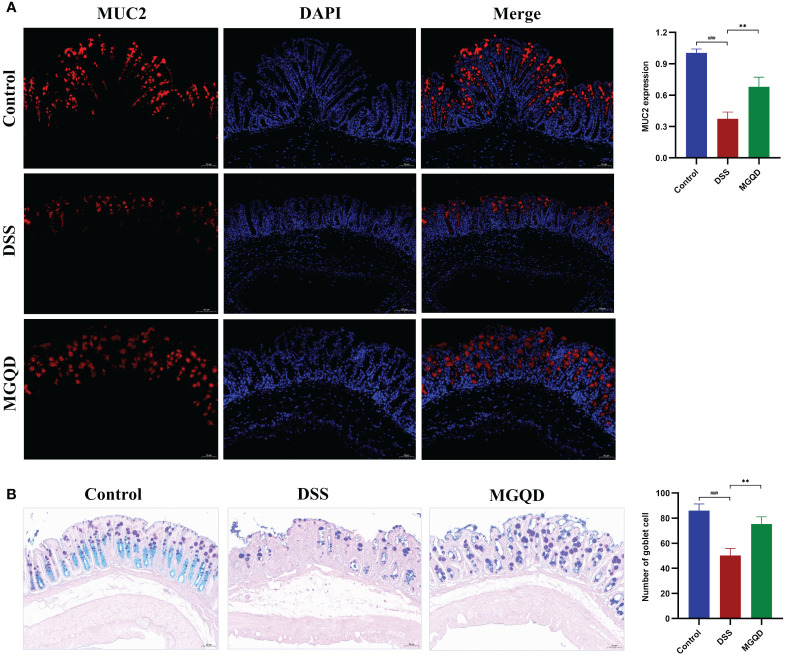
MGQD restored the intestinal mucus barrier by maintaining goblet cell function. **(A)** The expression of MUC2 protein via immunohistochemical analysis (200× magnification). **(B)** Representative AB/PAS-stained colonic tissue sections (200× magnification) and goblet cells number. ###*P* < 0.001 vs. control, **P < 0.01 vs. DSS.

The MUC2 protein is primarily responsible for forming the mucus barrier, which serves as the first defense of intestinal mucosal barrier. The results showed that MUC2 expression was downregulated in mice with UC but was reversed by MGQD treatment (**Figure 4A**). Goblet cells produce and release MUC2. According to AB/PAS staining, the number of goblet cells was decreased in mice with UC but was reversed by MGQD treatment (**Figure 4B**).

### MGQD increased the amount of Lrg5^+^ cells and stimulated cell proliferation

**Figure 5:**
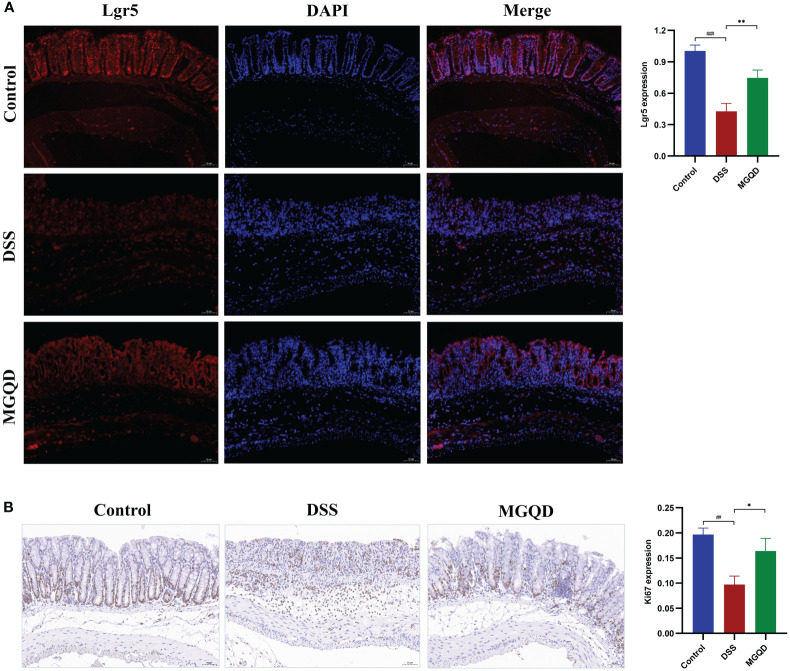
MGQD increased the amount of Lrg5^+^ cells and stimulated cell proliferation. **(A)** The expression of Lgr5 protein via immunohistochemical analysis (200× magnification). **(B)** Representative Ki67-stained colonic tissue sections (200× magnification). ##*P* < 0.01 vs. control, ###*P* < 0.001 vs. control, *P < 0.05 vs. DSS, **P < 0.01 vs. DSS.

Gut stem cells are identified by the expression of Lgr5, which is present in columnar cells at the base of intestinal crypts. The results showed that the number of Lgr5^+^ cells was decreased in mice with UC but was reversed by MGQD treatment (**Figure 5A**). Ki67 is a typical marker of cell proliferation. Our results showed that the number of Ki67^+^ cells was decreased in mice with UC but was reversed by MGQD treatment (**Figure 5B**). These findings imply that MGQD may ameliorate UC by stimulating intestinal epithelial cell regeneration via increased intestinal stem cell proliferation and differentiation inside the intestinal crypts.

### Gut microbiota might contribute to MGQD alleviating colitis in mice

**Figure 6:**
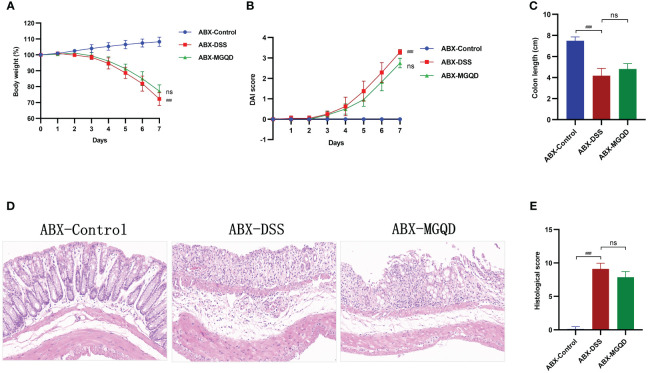
The protective effect of MGQD against colitis disappeared after gut microbiota depletion. **(A)** Daily changes in body weight in different groups, **(B)** daily changes in DAI score in different groups, **(C)** comparison of colon length, **(D, E)** representative H&E staining images of colon sections and the comparison of histopathological scores (200× magnification). ###*P* < 0.001 vs. control, ns *P* > 0.05 vs. DSS.

The gut microbiota was eradicated in mice using ABX cocktails. Compared with the ABX-control group, mice in the ABX-DSS group exhibited typical features of colitis, including weight loss, increased DAI, shortened colon length, and elevated colon histopathology score (**Figures 6A–E**). Surprisingly, mice with depleted gut microbiota did not experience relief of their colitis symptoms when treated with MGQD (**Figures 6A–E**).

**Figure 7:**
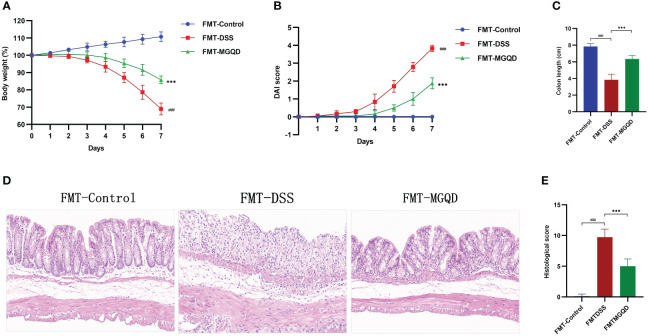
FMT ameliorated colitis induced by DSS. **(A)** Daily changes in body weight in different groups, **(B)** daily changes in DAI score in different groups, **(C)** comparison of colon length, **(D, E)** representative H&E staining images of colon sections and the comparison of histopathological scores (200× magnification). ###*P* < 0.001 vs. control, ***P < 0.001 vs. DSS.

We further validated the involvement of gut microbiota in the treatment of UC with MGQD by FMT experiments. Similarly, compared with the FMT-control group, mice in the FMT-DSS group exhibited typical features of colitis, including weight loss, increased DAI, shortened colon length, and elevated colon histopathology score (**Figures 7A–E**). However, when compared to the FMT-DSS group, mice in FMT-MGQD group showed significant improvement in both the above colitis symptoms and pathologic damage (**Figures 7A–E**).

### MGQD restored diversity and composition of gut microbiota

**Figure 8:**
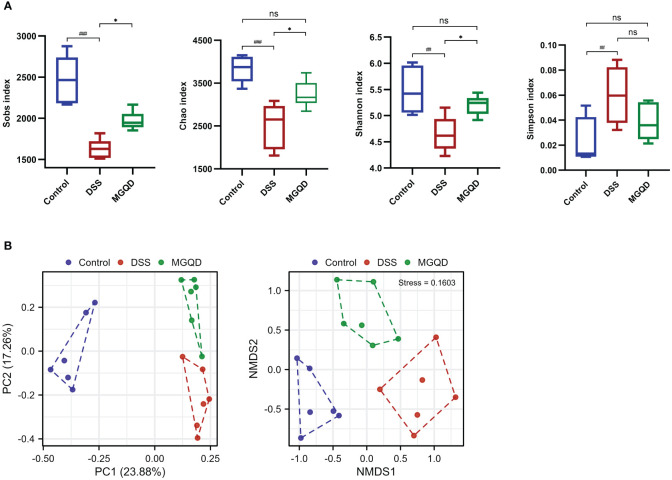
MGQD restored diversity of gut microbiota. **(A)** α-diversity analysis. **(B)** β-diversity analysis. ##*P* < 0.01 vs. control; ###*P* < 0.001 vs. control, *P < 0.05 vs. DSS, ns *P* > 0.05. P < 0.01 vs. control.

Following a similar trend by Sobs, Chao, Shannon, and Simpson, we found that gut microbial α -diversity was significantly decreased in UC mice but reversed by MGQD treatment (**Figure 8A**). Furthermore, the microbiomes of the MGQD treated mice were distinctly different from the DSS alone treated mice using non-metric multi-dimensional scaling and principal co-ordinates (**Figure 8B**).

**Figure 9:**
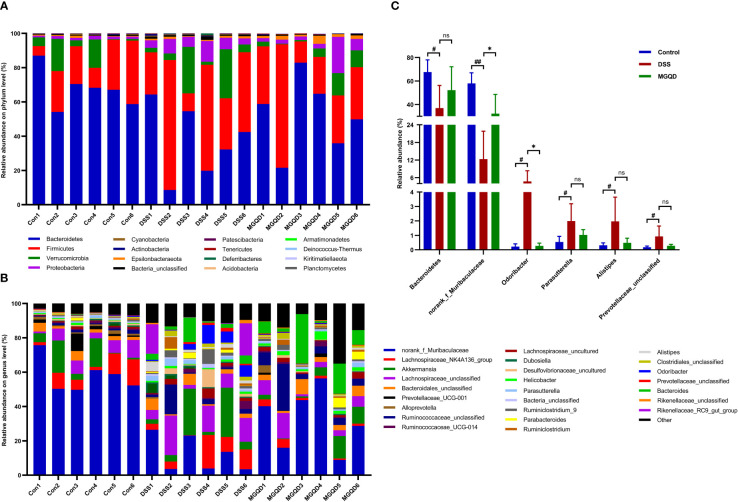
MGQD restored composition of gut microbiota. **(A)** The composition of gut microbiota at the phylum level. **(B)** The composition of gut microbiota at the genus level. **(C)** Phylum and genera that were statistically different with the treatment of DSS and MGQD. In this section, results that were not statistically different between any of the groups were not shown. #*P* < 0.05 vs. control, ##*P* < 0.01 vs. control, *P < 0.05 vs. DSS, ns *P* > 0.05.

At phylum level (**Figure 9A**), the structure of gut microbiota in all groups of mice was dominated by *Firmicutes*, *Verrucomicrobia, Bacteroidetes* and *Proteobacteria*. Our results (**Figure 9C**) showed that b *Bacteroidetes* were significantly reduced in colitis mice compared to mice in the control group (*P* < 0.05). Furthermore, *Bacteroidetes* were increased in mice treated with MGQD compared with mice in the DSS group, but there was no statistical difference (**Figure 9C**). At genus level (**Figures 9B, C**), *norank_f_Muribaculaceae* was reduced (*P* < 0.05), while *Odoribacter*, *Parasutterella*, *Alistipes* and *Prevotellaceae_unclassified* was increased in colitis mice (*P* < 0.05). However, there was an increase in *norank_f_Muribaculaceae* (*P* < 0.05) and a reduce in *Odoribacter* (*P* < 0.05) in mice treated with MGQD compared to the DSS group.

**Figure 10:**
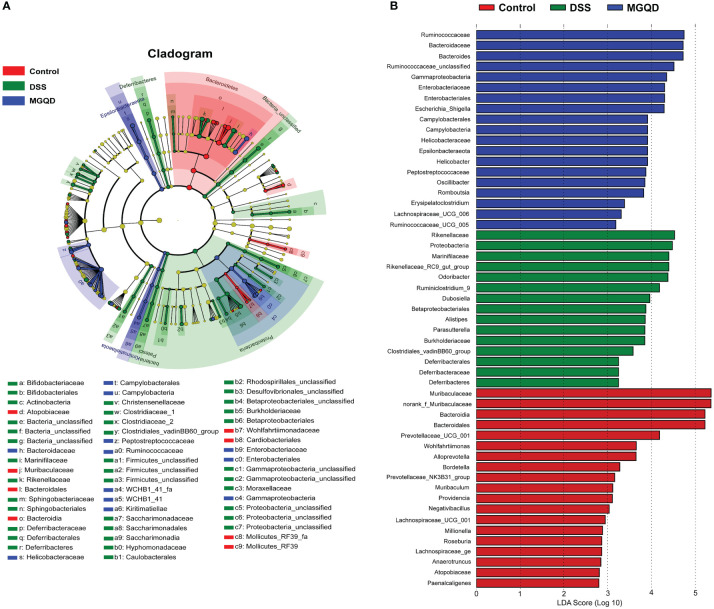
Difference in dominant microorganisms among three groups via cladogram and distribution histogram based on LDA. **(A)** Cladograms generated from the LEfSe analysis, showing the variation of microbial compositions at the phylum, class, order, family, and genus level. **(B)** LDA scores derived from LEfSe analysis, showing the biomarker taxa.

Results of Lefse (**Figure 10**) showed that *Proteobacteria* (from phylum to family) and *Deferribacteres* (from phylum to family) were the key bacterial types contributing to the imbalance of gut microbiota in mice with UC. Mice in the MGQD group were dominated at the phylum level by *Gammaproteobacteria* and *Campylobacteria*, at the order level by *Enterobacteriales* and *Campylobacterales*, and at the family level by *Enterobacteriaceae*, *Helicobacteraceae*, *Bacteroidaceae*, *Ruminococcaceae* and *Peptostreptococcaceae*, and these bacterial types may be corrected with MGQD-mediated attenuation of UC.

### MGQD treatment improved BA metabolism

**Figure 11:**
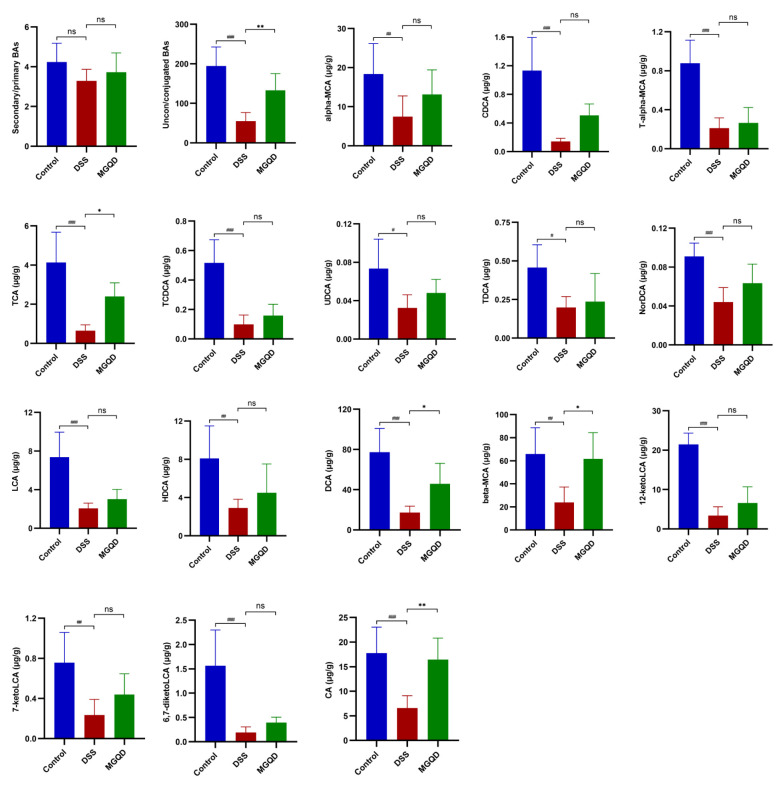
BA metabolism is regulated by MGQD. # *P* < 0.05 vs. control, ## *P* < 0.01 vs. control, ###*P* < 0.001 vs. control, * *P* < 0.05 vs. DSS, ** *P* < 0.01 vs. DSS, *** *P* < 0.001 vs. DSS, ns *P* > 0.05 vs. DSS.

Our results (**Figure 11**) suggested that the ratio of unconjugated BAs to conjugated BAs in the feces of UC mice was drastically decreased (*P* < 0.05), but reversed by MGQD treatment. In addition, the levels of α-MCA, CDCA, T-α-MCA, TCA, TCDCA, UDCA, TDCA, NorDCA, LCA, HDCA, DCA, β-MCA, 7-ketoLCA, 12-ketoLCA, 6,7-diketoLCA and CA in mice with UC were reduced (*P* < 0.05) compared to mice in control group. Interestingly, levels of TCA, DCA, β-MCA, and CA were increased in the MGQD-treated mice compared to the DSS group (*P* < 0.05).

**Figure 12:**
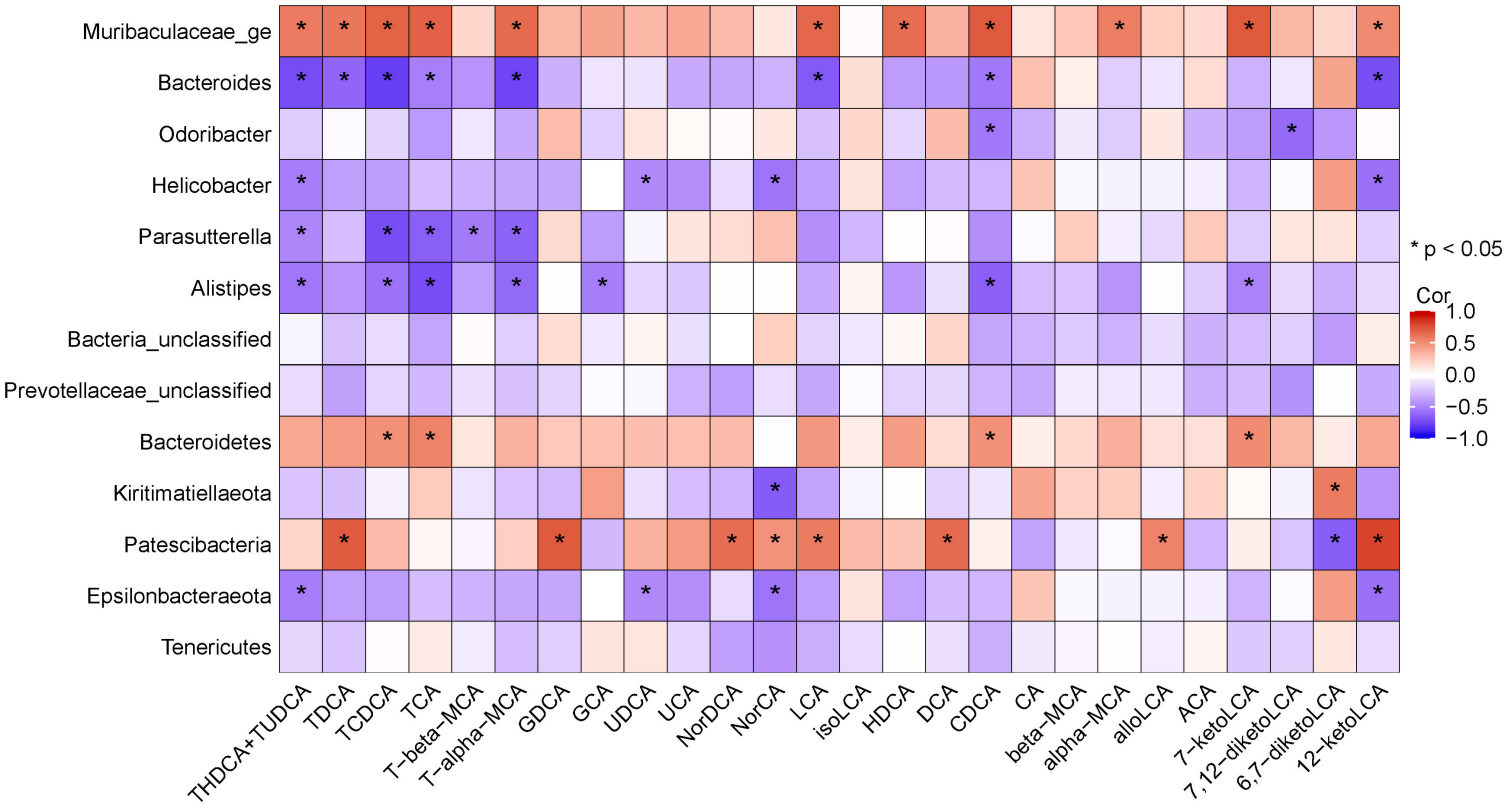
Heatmap showing Spearman’ correlation coefficient between several significant changed BAs and bacteria.

The potential association between altered gut microbiota and BAs was determined using Spearman correlation analysis. The results (**Figure 12**) suggested that *Bacteroides* was positively linked with TCDCA, TCA, CDCA, 7-ketoLCA. *Norank_f_Muribaculaceae* was positively correlated with TDCA, TCDCA, TCA, T-α-MCA, LCA, HDCA, CDCA, α-MCA, 7-ketoLCA, and 12- ketoLCA. *Odoribacter* was negatively linked with CDCA. *Alistipes* was negatively linked with TCDCA, TCA, T-α-MCA, CDCA and 7-ketoLCA. *Parasutterella* was negatively correlated with TCDCA, TCA and T-α-MCA.

## Discussion

Based on the DSS-induced UC mouse model, we gave MGQD continuous intervention for 7 days to evaluate its therapeutic effect. It was found that mice treated with MGQD had significantly reduced colitis symptoms and colonic pathologic damage compared to mice in DSS group. Measurements of inflammatory cytokine levels also suggested that the levels of IL-1β, TNF-α and IL-6 in the colonic tissues of mice intervened by MGQD were decreased compared with those of DSS group, suggesting that MGQD helps to inhibit intestinal inflammatory responses in mice with UC. In addition, MGQD was found to restore the function of t function of goblet cells and intestinal mucus barrier by promoting the proliferation and differentiation of intestinal stem cells (ISCs) as well as the secretion of MUC2 protein, respectively. Most importantly, we showed that MGQD reduced colonic damage in a gut microbiota-dependent way using intestinal bacterial depletion and FMT experiments.

Accumulating evidence suggests that patients with UC have a decreased diversity of gut microbiota, mainly characterized by an increase in conditionally pathogenic and harmful bacteria and a decrease in beneficial bacteria and (23–25). Our results indicated that mice with UC presented decreased microbial diversity and decreased *Bacteroidetes and norank_f_Muribaculaceae* and increased *Odoribacter*, *Parasutterella*, *Alistipes* and *Prevotellaceae_unclassified*. It is noteworthy that *Odoribacter*, *Alistipes* and *Prevotellaceae_unclassified* are all classified under *Bacteroidetes*. According to earlier research, gut microbiota of individuals with UC suffers structural damage due to reduced diversity of *Bacteroidetes*, and that *Bacteroides* were correlated with disease activity (26, 27). Therefore, the altered diversity of *Bacteroidetes* has been considered as a marker of gut microbiota dysbiosis in UC (28). Consistent with the reports of previous studies (29–31), the present study found that *norank_f_muribaculaceae*, which accounted for the largest proportion of mice in each group and are beneficial for maintaining intestinal health, were reduced significantly in mice with UC. Interestingly, both *Bacteroidetes* and *norank_f_muribaculaceae* was restored in the MGQD-intervened mice compared to mice in DSS group.

In addition to the gut microbiota and their function, there may be other factors involved in the treatment of UC with MGQD. Immune-related factors should be the first focus of attention, as a broadly dysregulated immune response is critical in the pathologic process of UC (32). Targeting immune cell circuits and cytokines has been used as a rational basis for new translational therapies for inflammatory bowel disease (33). Previously published studies have reported that MGQD has a regulatory effect on Treg/Th17 balance and activation of γδT17 cells (20, 34). Oxidative stress has long been known to be a non-negligible causative factor in the inflammation of mucosal tissues in patients with UC, and targeting oxidative stress may provide an exciting avenue for combating mucosal inflammation (35). Previous studies suggested that Gegen Qinlian decoction could modulate Nrf2/ARE signaling and enhance antioxidant effects to alleviate experimental colitis (36, 37). Biological barrier, chemical barrier, mechanical barrier and immune barrier cooperate to maintain the integrity of intestinal mucosal barrier, which is closely related to intestinal health. Probiotics have been reported to improve the intestinal mucosal barrier and alleviate the inflammatory response by synergistically activating the biological, chemical, mechanical and immune barriers (38). Similarly, Gegen Qinlian decoction and MGQD were found to improve the intestinal mucosal barrier by repairing the mechanical barrier (18, 19, 39). Programmed cell death is a new hotspot that has been reported to be involved in the pathological process of UC in recent years (7), and natural compounds possess the potential to target programmed cell death signaling mechanisms to alleviate UC (40). Recent studies have found that Gegen Qinlian decoction and MGQD show potential for targeting ferroptosis and disulfidptosis to alleviate experimental colitis (19, 39, 41). Environmental factors have also been implicated as important factors in inducing the development and progression of UC (42). Interestingly, a study suggests that the main active components of Jiawei Gegen Qinlian decoction has different mitigating effects on colitis mice under different dietary environments, suggesting that environmental factors may also be involved in the mitigation of UC by Gegen Qinlian decoction (43).

Gut microbe-host interactions are often mediated through gut microbe metabolites (44, 45). Accumulating evidence suggests that imbalances in BA metabolism are associated with UC (46). In the present study, the levels of α-MCA, CDCA, T-α-MCA, TCA, TCDCA, UDCA, TDCA, NorDCA, LCA, HDCA, DCA, β-MCA, 7-ketoLCA, 12-ketoLCA, 6,7-diketoLCA and CA in mice with UC were reduced compared to mice in control group. Interestingly, levels of TCA, DCA, β-MCA, and CA were increased in the MGQD-treated mice compared to the DSS group. Similarly, plasma levels of TUDCA, TCDCA, CDCA, β-MCA, and DCA were reported to be significantly decreased in UC mice compared with normal mice, whereas the levels of TUDCA, TCDCA were significantly increased after treatment with Sijunzi decoction (47). In addition, it has been reported that the contents of CA and ωMCA decreased and the level of CA increased in feces of UC mice (48). Interestingly, level of α-MCA was reversed after MGQD treatment, while levels of CA and ωMCA were further increased. Moreover, it has been reported that the contents of DCA, UDCA, T-DCA, and DCA decreased and the level of CA increased in serum of UC mice (49). Surprisingly, these disturbed BA levels were reversed after treatment with Qingchang Huashi Formula. It is important to note that the BA metabolic profiles of mice with experimental colitis identified in different studies are not always consistent or even paradoxical, which may be associated with differences in modeling details, sample differences, and differences in detection methods. Despite the differences in the results of these studies, they all consistently imply that the Chinese herbal compound has a positive regulatory effect on BA metabolism imbalance in mice with UC.

Germ-free animals are regarded as a “golden model” to explore the causal relationship between gut microbiota and disease (50, 51). However, it is challenging to use germ-free animals to carry out experiments in general laboratories due to factors such as difficult access, high price, and strict feeding environment requirements. Multiple broad-spectrum ABX “cocktails” have been reported to deplete more than 90% of gut microbes in mice and pseudo germ-free mouse models close to germ-free mouse standards can be obtained (52). Therefore, most investigators tend to choose pseudo germ-free mice instead of germ-free mice to carry out studies related to gut microbiota (53, 54). According to a previous study, germ-free and antibiotic-treated mice are highly susceptible to epithelial injury in DSS colitis compared to normal mice (55). Therefore, we used the quadruple ABX “cocktails” to construct a pseudo germ-free mouse model based on previous experience in this experiment (20). In the present study, we found a loss of alleviation of colitis in pseudo germ-free mice by MGQD. By FMT, we found that pseudo germ-free mice receiving microbiota intervened by MGQD showed significant colitis remission compared to pseudo germ-free mice receiving microbiota intervened by DSS, suggesting the possible involvement of gut microbiota in the treatment of UC by MGQD. Our findings are consistent with several previous studies (56–59) reporting that the efficacy of herbal compounds is weakened or even eliminated in the presence of depleted gut microbiota, while FMT can transfer the effect of herbal compounds to the gut microbiota to exert disease-improving efficacy.

Limitations should be acknowledged. First, while this study identifies gut microbiota that may be involved in the amelioration of UC by MGQD, there may be other factors involved that could not be identified. Second, the regulatory effect of Chinese herbal decoction on ISCs in acute and chronic UC models may be bidirectional (60), so further exploration of the proliferation status of ISCs and the effect of MGQD on them based on chronic UC models in the future is more conducive to a comprehensive understanding of the comprehensive prevention and treatment mechanism of MGQD on UC. Third, the lack of evaluation of anti-inflammatory cytokines such as TGF‐β and sfTSLP in this study makes further evaluation of the effect of MGQD on anti-inflammatory cytokines still necessary in the future.

## Conclusion

MGQD significantly alleviated colitis in mice, which may be associated with the modulation of gut microbiota and BA metabolism and restoration of function of goblet cells. However, factors other than the gut microbiota may also be involved in the amelioration of UC by MGQD.

## Data availability statement

Sequencing data of the present study are provided in: http://www.ncbi.nlm.nih.gov/bioproject/1140514 (BioProject ID: PRJNA1140514).

## Ethics statement

The animal study was approved by Animal Ethics Committee of Xi Yuan Hospital of China Academy of Chinese Medical Sciences (Approval NO. 2019XLC003-2). The study was conducted in accordance with the local legislation and institutional requirements.

## Author contributions

JH: Writing – original draft, Methodology, Data curation, Conceptualization. JZ: Writing – original draft, Methodology, Data curation. FW: Writing – original draft, Methodology, Data curation, Conceptualization. XT: Writing – review & editing, Writing – original draft, Supervision, Methodology, Data curation.
